# Reply: Re-evaluating Post-TAVR Antithrombotic Strategies in Nonanticoagulated Patients

**DOI:** 10.1016/j.jacadv.2025.101979

**Published:** 2025-07-07

**Authors:** Sahand Siami, Sina Kazemian, Saba Maleki, Sara Ebrahimi, Kaveh Hosseini

**Affiliations:** a Tehran Heart Center, Cardiovascular Diseases Research Institute, Tehran University of Medical Sciences, Tehran, Iran; b The University of Texas MD Anderson Cancer Center, Houston, Texas, USA

We thank Dr Skalidis and colleagues for their insightful commentary and the opportunity to further discuss our findings on the recent study.^1^

First, we agree that the modest differences in ischemic outcomes across regimens highlight the relatively low residual thrombotic risk in this population. In our analysis, more intensive strategies, including dual antiplatelet therapy and oral anticoagulant (OAC)-based regimens, did not reduce thrombotic events compared with single antiplatelet therapy (SAPT) but were associated with higher bleeding risk, suggesting that net clinical benefit is driven by safety rather than improved thrombotic risk. While some studies have linked OAC use to reductions in subclinical leaflet thrombosis, as detected by cardiac computed tomography, this surrogate marker has not been associated with hard clinical outcomes. However, individualized use of OAC-based regimens in patients with subclinical leaflet thrombosis confirmed by cardiac computed tomography may help reduce the bleeding risk.^2^

Second, as mentioned by Dr Skalidis and colleagues, low-dose rivaroxaban plus SAPT was associated with increased all-cause mortality in our analysis, which was primarily driven by noncardiovascular causes beyond the treatment window. A similar trend in the ATLANTIS trial with apixaban suggests a potential class effect of direct OACs (DOACs) in frail patients, independent of dosing. However, this pattern has not been consistent across all DOACs. A meta-analysis by Al Said et al reported comparable survival between edoxaban and antiplatelet therapy in nonindicated patients.^3^ Future trials need to focus on drug-specific risks, dosing, and patient characteristics.

Third, our meta-regression identified chronic obstructive pulmonary disease as a comorbidity significantly modifying bleeding risk with SAPT vs dual antiplatelet therapy. This supports the recognition that extracardiac factors can influence outcomes. While valve type may affect procedural risk, comorbidity-based stratification allows tailoring therapy. Tools like BLeNet have demonstrated feasibility in integrating chronic obstructive pulmonary disease and renal function into bleeding risk models.^4^ However, prospective validation is needed to assess whether these strategies improve decision-making.

Finally, we appreciate the suggestion regarding valve-specific stratification. We think characteristics such as radial force and leaflet thrombosis may influence thrombotic and bleeding risks.^5^ However, in our data set, approximately 70% of patients received balloon-expandable valves, limiting stratified analysis. Future studies with prespecified comparisons are warranted. In conclusion, SAPT appears to offer the most favorable safety-efficacy profile, but further research is required to clarify the role of DOACs and potential individualized approaches.

## References

[1] Siami S., Kazemian S., Maleki S. (2025). Post-transcatheter aortic valve replacement antithrombotic treatment in nonindicated patients. JACC Adv.

[2] Capodanno D., Collet J.-P., Dangas G. (2021). Antithrombotic therapy after transcatheter aortic valve replacement. JACC Cardiovasc Interv.

[3] Al Said S., Kaier K., Nury E. (2025). Non-vitamin K antagonist oral anticoagulants (NOACs) after transcatheter aortic valve replacement (TAVR): a network meta-analysis. Cochrane Database Syst Rev.

[4] Jia Y., Luosang G., Li Y. (2022). Deep learning in prediction of late major bleeding after transcatheter aortic valve replacement. Clin Epidemiol.

[5] Bhogal S., Waksman R., Shea C. (2024). Self-expanding and balloon-expandable valves in low risk TAVR patients. Int J Cardiol.

